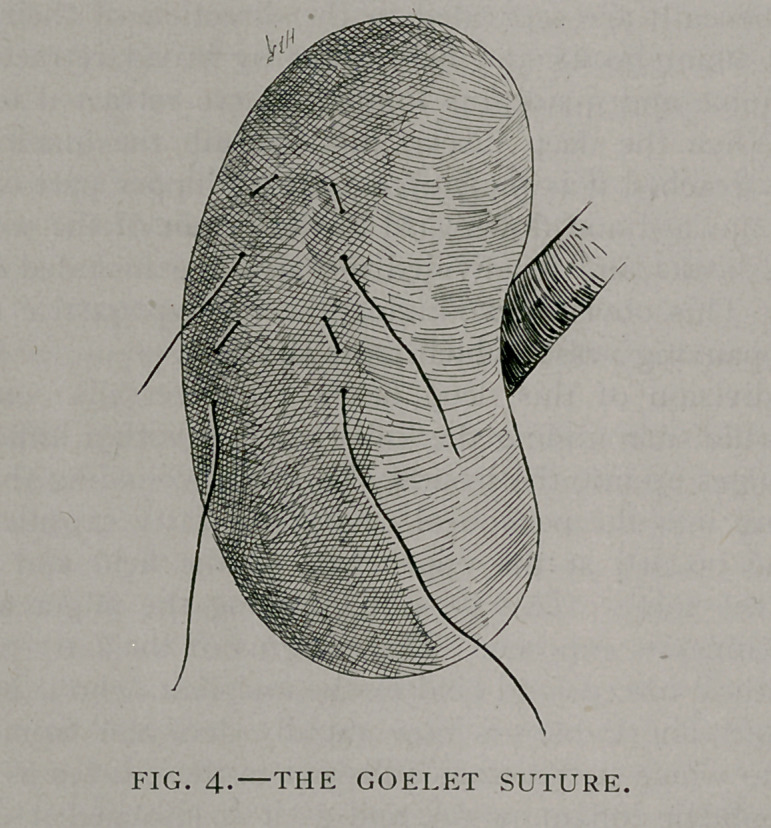# Diagnostic Palpation of Prolapse of the Kidney and Method of Fixation1Presented by invitation at the Fourth Annual Meeting of the Lake Keuka Medical and Surgical Association, August 11 and 12, 1903.

**Published:** 1903-11

**Authors:** Augustin H. Goelet

**Affiliations:** Professor of gynecology, New York School of Clinical Medicine; gynecological surgeon to Metropolitan Hospital for Women and Children, New York, etc.


### Diagnostic Palpation of Prolapse of the Kidney and Method of Fixation.[1]

By AUGUSTIN H. GOELET, M. D.

Professor of gynecology, New York School of Clinical Medicine; gynecological surgeon to Metropolitan Hospital for Women and Children, New York, etc.

THE importance of prolapse of the kidney to the general practitioner is to be estimated by the frequency with which it is met with, particularly in women, and the diversity of the symptoms it gives rise to which are very misleading and are often attributed to other cause. It is generally conceded that it is to be found in 20 per cent. of all women or one in every five, and I have found by carefully prepared statistics in my clinic that it occurs in from 30 to 35 per cent. of all gynecological patients. It occurs seven times more frequently in women than in men, but it has been observed so often among street-car employees in the west that it has been designated "railroad kidney."

The question naturally arises when are we to suspect this condition and in what cases are we to look for it? I contend that all gynecological patients should be examined for it and that the examination should be instituted first while the patient is on her

---

1. Presented by invitation at the Fourth Annual Meeting of the Lake Keuka Medical and Surgical Association, August 11 and 12, 1903.

feet before she is placed in the recumbent position for the pelvic examination. This is essential because the kidney becomes replaced, as a rule, when the patient assumes the recumbent position, and is not always displaced again immediately upon resumption of the erect position; often not until the patient has been on her feet for a while or has become fatigued. I am sure that many of these prolapsed kidneys are overlooked because this rule is not followed, or because the examination is only made when the patient is in the recumbent position, for it is more readily detected when the patient is standing.

The symptoms found associated with prolapse of the kidney and which may be attributed to it, because they are overcome by fixing the kidney, or temporarily relieved many times by adjusting effective support for the organ by external pressure upon the abdomen, may be grouped under three general heads,[2], viz.:

1. Those denoting disturbance of the digestive organs, manifested by irritability of the stomach, loss of appetite, intestinal distention, constipation, recurring bilious attacks, with sometimes transient attacks of jaundice.

2. Those relating to disturbance of the circulation and nervous system, manifested by vertigo, syncope, palpitation of the heart, epigastric pain, insomnia, lassitude or unusual fatigue upon exertion, with hysteria and neurasthenia.

3. Those denoting disturbance of the genitourinary organs, manifested by diseases of the female pelvic organs, that may result from prolonged congestion, caused by pressure of the prolapsed kidney upon the ovarian vein, hence obstruction of the return circulation from the pelvis[3], irritability of the bladder, which sometimes results in cystitis, and renal disturbances, such as result from interference with the circulation of the kidney and obstruction to the outflow of excreted urine from the kidney[4].

### METHODS OF PALPATION AS A MEANS TO DIAGNOSIS.

I shall describe only those methods of palpation that I have found most satisfactory after an abundant experience extending over a period of several years. The erect position is best adapted for detecting the kidney when displaced, but in some cases, especi-

---

2. These symptoms may be found described in detail in an article by the author entitled, "Diagnosis of prolapsed kidney, with illustrations of the method of diagnostic palpation", in the *International Medical Magazine* for June, 1902.

3. For an explanation of the Influence of prolapse of the kidney in producing female pelvic disease, the reader is referred to an article by the author in the *Journal of the American Medical Association,* August 23, 1902.

4. For explanation of the effect of prolapse on the kidney itself, the reader is refered to an article by the author entitled, "A study of the indications for nephropexy", in the *Medical Record,* December 20, 1902, and the *New York State Journal of Medicine,* July, 1903. Also the *Philadelphia Medical Journal,* September 6, 1902.

ally where there is rigidity of the abdominal wall, the recumbent position with the shoulders raised, as will be described below, is more conducive to satisfactory palpation, and in all cases when the kidney is not revealed in the erect position the patient should also be examined in the dorsal position. I have repeatedly emphasised the necessity of palpating for the kidney with the patient in the erect position before making the examination of the pelvis, because a kidney that may be considerably prolapsed when the patient has been walking or standing or even sitting, becomes replaced as soon as the patient is placed on her back and often it is not easily displaced even after she gets on her feet again. It is also difficult or even impossible many times to detect the

FIG. I. GOELET—PALPATION.

minor degrees of prolapse (first and second degrees) when the patient is in the dorsal position.

To palpate the kidney in the erect position, the clothing is loosened about the waist, the corset being removed, and the abdomen bared from the border of the ribs to below the level of the umbilicus. The patient is made to stand erect upon both feet with the sacrum resting against a table not too low, a door, or the wall, so as to fix the pelvis, and she is made to bend forward with the pelvis fixed, thus flexing the spine on the pelvis which will secure relaxation of the abdominal wall. To still further favor relaxation of the abdominal wall she may stand on the opposite foot and rest that of the side being examined upon the toe. The examiner sits in front of her and if it is the right side that is to be palpated, he grasps the loin with the left hand, the fingers behind, with the middle finger at the level of the lower border of the last rib behind and the thumb in front at the level of the lower border of the last rib in front. (See illustration, Fig. 1). The patient is now directed to take a deep inspiration and to expire slowly to the extreme limit. Upon inspiration no pressure is to be exerted by the hand grasping the loin, but upon expiration compression is to be made and maintained after it is complete, the patient being directed not to breathe again for a moment, or if it becomes necessary, not to breathe deeply. This grasp of the loin below the ribs, narrows the space through which the kidney must move and fixes it for palpation if it is prolapsed so that its upper pole descends below the border of the last rib in front (third degree).

Maintaining this pressure of the left hand upon the loin, with the thumb pressed well under the rib, the abdomen below is palpated with the right hand, as shown in Fig. 2. In this manner,

FIG. 2. GOELET—PALPATION.

if the kidney is prolapsed, the lower pole is caught by the finger tips of the right hand on the abdomen and the upper pole is forced up against the thumb of the left hand depressing the abdominal wall, just below the border of the rib above. Thus the size and location of the kidney can be readily made out if the abdominal wall is sufficiently thin or relaxed. To ascertain definitely that this is the kidney, the compression of the loin by the left hand is slightly relaxed and pressure upon the lower pole by the right hand causes it to slip up into position and as it slips upward between the thumb and finger of the left hand it can be distinctly outlined. If now the patient is directed to take a deep inspiration again while the grasp of the loin is only slightly relaxed, just sufficiently to permit the kidney to slip between the thumb in front and finger behind, it can be felt again as it descends.

The minor degrees of descent (first and second degrees), can be made out only by this latter maneuver, the lower pole or lower half of the organ being grasped between the thumb and finger compressing the loin upon deep inspiration, but the kidney slips up and back into position when it is compressed.

If palpation is to be made for the left kidney, which is less frequently prolapsed than the right, the hands are reversed, the left loin being grasped with the right hand and the abdomen palpated with the left.

For palpation with the patient in the dorsal position, the manipulation is somewhat different. The patient is placed upon the back with a roll under the shoulders, over which the upper spine is flexed backward with the head touching the table or

FIG. 3. GOELET—PALPATION.

couch and with the thigh of the side to be examined flexed upon the abdomen and the foot resting in an easy position upon the table. This position bows the lumbar spine forward, throwing the kidney forward within easy reach, and effects complete relaxation of the abdominal wall. The examiner standing to the right for examination of the right kidney, places the left hand upon the lumbar region and with the middle finger depresses the lumbar region upward, just below the border of the last rib. Then, with the finger-tips of the right hand upon the abdomen at the lower border of the last rib in front, he directs the patient to take a deep inspiration, which displaces the kidney, if it is movable, and as the patient expires he forces the fingers deeply under the rib, depressing the abdominal wall. If the kidney descends to the third degree it is caught above its upper pole. (See Fig. 3).

If now he makes the thumb of his left hand take the place of his right, as in making the examination in the erect position, he can hold the kidney displaced while his right hand is free for manipulation of the abdomen and palpation of the kidney.

One thing must be kept constantly in mind, *i. e.*, that it is essential to maintain the pressure of both the middle finger behind and thumb in front, so as to prevent the kidney from slipping back into position.

If the patient is nervous and the abdominal wall is rigid, satisfactory palpation is impossible. In such cases repeated examinations with intervals of rest is the only remedy. If the colon is distended it will frequently be impossible also to make out even a badly prolapsed kidney until the bowel has been thoroughly emptied by a cathartic or enema or both. When prolapse of the kidney is suspected and the examiner is in doubt it is wiser to request and make another examination under more favorable conditions rather than give a negative or doubtful opinion, since the next man to whom the patient goes for advice may discover it and apprise her of your error.

Examination under anesthesia may be called for in some cases, but is rarely necessary and is, as a rule, unsatisfactory, because of the position of the patient, and because being unconscious she cannot aid in dislodging the kidney.

*Differential Diagnosis.*—Having discovered a movable mass where the displaced kidney should be, there are several conditions from which it must be differentiated, viz.:

(1) A distended gall-bladder; (2) tumor of the pylorus; (3) tumor of the omentum; (4) growths in the intestine, particularly in the colon at the hepatic or splenic flexure; (5) impacted feces.

The distended gall-bladder usually lies more anteriorally than the prolapsed kidney and often forms a visible tumor, whereas the latter never appears as a visible tumor, unless the kidney is greatly enlarged and the abdominal wall is very thin. If the distended gall-bladder is displaced upward by pressure it moves directly upward and does not disappear upward and backward as does the kidney. If the tumor is a distended gall-bladder it reappears when the patient is in the recumbent position, whereas the kidney, as a rule, remains replaced in this position or is not dislodged except on deep inspiration.

Pressure upon the distended gall-bladder usually gives rise to considerable pain, while pressure upon the prolapsed kidney does not produce pain, though it may cause a sickening sensation, or the patient may flinch, because the kidney is sensitive or sore.

The chief characteristics of the prolapsed kidney is the facility with which it may be replaced, particularly when the patient is on her back, and its range of mobility is frequently considerable, but the distended gall has a limited range of mobility and is not readily displaced upward. The kidney, however, sometimes becomes fixed in its abnormal position of descent as the result of perinephritic inflammation and cannot be replaced while at the same time it may not be wholly immovable. In other cases the kidney may be greatly enlarged as the result of turgescence, hydronephrosis or pyonephrosis or exudation and induration of the surrounding fat and form a very distinct tumor, too large to occupy its original position. In such cases there must often be an element of doubt in the diagnosis.

Tumors of the pylorus are nearly always associated with symptoms of obstruction and it is seldom that they can be made to disappear by palpation. When displaced by pressure they move directly upward and not upward and backward as does the kidney.

Tumors of the omentum are usually malignant and there is generally to be found evidence of malignancy elsewhere in the abdomen.

Growths in the colon, particularly those near the hepatic flexure, may be mistaken for the kidney, but repeated examinations in different positions, or distention of the colon with air or gas, should clear up the diagnosis. Anesthesia would aid very materially in making a diagnosis in these cases, but the bowel should be empty at first and distended afterwards if necessary.

Impacted feces making a tumor in this location can be removed by clearing out the bowel.

*Indications for Operation.*—Inasmuch as the kidney prolapsed to the third degree or beyond (that is, with its upper pole below the border of the last rib in front), cannot be effectively and safely maintained in its normal position by any means of external support, whether by belt or corset, and inasmuch as such support is often productive of serious injury to the kidney from the pressure it exerts, it may be said that prolapse of the organ to the third degree or beyond is indication for operation to secure its fixation in normal position.

I have often heard it asserted that there are many women walking about with their kidneys prolapsed, who would never know it if they were not told of it. This is very true and their symptoms are attributed to other cause and treated accordingly. There was never a greater mistake made than to suppose that such patients are free from the risks incurred by neglected prolapse of this organ. Incipient inflammation of the kidney may be found in many of these women walking around with undiscovered prolapse of the kidney, which could be detected only by submitting the urine to careful, expert microscopical examination. I have found by careful analysis that 75 per cent. of the patients with prolapse of the kidney to the third degree who wear corsets have either a mild pyelonephritis or interstitial nephritis. It is well known that in addition to nephritis and pyelonephritis, hydronephrosis, pyonephrosis and atrophy of the organ may result from long-continued prolapse, because of interference with its circulation and draining off of the excreted urine.

But the indications for the operation are to be found:

1. In the disturbance or inconvenience arising from the prolapse which in many instances produces chronic invalidism.

2. The influence of the prolapse in producing disease of the female pelvic organs, which is not overcome except by fixing the kidney in its normal position.

3. The influence of the prolapse on the kidney itself, whereby its function is seriously interfered with, rendering inflammation of its structure constantly imminent. The prolapsed kidney must therefore be regarded as a crippled organ deserving, in consequence of its importance to the human economy, our best consideration.

If I hold, apparently, extreme views upon the indication for operation in these cases, it is because I have seen so often the folly of delay and the inefficiency and harmfulness of other attempts at relief. We have in prolapse of the kidney a condition that is inevitably progressive and one which seriously interferes with the circulation and function of the organ and the health of the patient; one which must eventually lead to serious disease of the kidney, and there can be no valid excuse for withholding an operation which involves no risk whatever.

*Method of Fixation.*—It is possible to secure satisfactory and permanent fixation of the kidney in its normal position without injury to its structure and without depriving it of its fibrous capsule. To accomplish this the attached colon must be completely detached to obviate subsequent dragging upon the kidney, which would either tear it away from its attachment or cause constant pain. The sutures must be inserted under the fibrous capsule only in such manner that they will not tear out, and they must be tied so that the suture loop will not loosen and permit the kidney to sag away from contact with the structures to which it is desired to have it adhere. In other words, close, uninterrupted contact of the kidney to the structures of the back until adhesion can occur is absolutely essential for success. Stripping off the fibrous capsule is by no means necessary and is objectionable because it inflicts an injury that may prove harmful. Cases have been reported where atrophy of the organ followed this procedure.

The patient is placed prone upon the table upon the chest, with a roll or rubber air-pillow eight inches in diameter under the abdomen. The operator stands on the patient's left for operating on both kidneys.

A vertical incision is made along the outer border of the erector spinæ muscle from the last rib downward for about three or three and a half inches. This incision is carried down through the fat underlying the integument to the aponeurosis covering the first layer of muscles. This is divided with scissors and the muscles beneath are separated in the direction of their fibers by means of blunt hooks and held apart by broad retractors. The erector spinæ and quadratus lumborum are retracted toward the spine. When the deep fascia, just beneath the quadratus lumborum, is reached it is punctured near the upper part of the field and split upward and downward to the extent of the wound with the blunt hooks and its divided margins are included in the retractors. This obviates wounding the ileohypogastric nerve and its accompanying vessel which cross the field.

The division of this deep fascia (transversalis) exposes the fatty capsule surrounding the kidney and it with a knuckle of intestine bulges up into the wound. To avoid wounding the intestine or opening into the peritoneal cavity, the fatty capsule is drawn down and opened at the upper part of the field and well over towards the spine. This is split up along the finger as a guide and the kidney is exposed. The margins of the fatty capsule are seized with T forceps and held by the assistant, who is holding the retractors. The kidney is now rapidly detached from the fatty capsule, to which it is always adherent in these cases as the result of perinephritic inflammation, and as it is loosened it is dragged upon and delivered through the incision on to the surface of the back. To facilitate this the lower pole of the kidney is freed first and brought up into the wound by traction upon the fatty capsule, and at the same time the patient is rolled downward upon the air-pillow so as to bring its pressure upon the lower border of the ribs which tends to force the kidney downward. To facilitate still more the delivery of the kidney it is seized with a piece of gauze held between the thumb and finger and dragged upon, but it should not be handled roughly. Care is taken now to free the kidney both upon its anterior and posterior faces quite around to the hilum and both poles, thus completely separating the attached colon in front. Where the detachment cannot be readily accomplished with the fingers scissors are used to divide unyielding constricting bands. The ureter and pelvis of kidney are palpated carefully for stone, which if found are removed.

We are now ready for insertion of the sutures which are to hold the kidney in position until it can become firmly attached by adhesion. Two sutures are employed and the material used is silkworm gut. These are inserted in the manner shown in the accompanying illustration (Fig. 4): one at the junction of the middle with the lower third of the organ and the other at about its center and upon the posterior aspect of the free border of the kidney. These sutures are inserted only under the fibrous capsule which is left intact and both have three insertions. A

FIG. 4.—THE GOELET SUTURE.

very fine curved needle is used for carrying the sutures which are drawn through carefully so as to avoid tearing the capsule.

The redundant fatty capsule is removed preferably by tearing it away, care being taken to push the intestine back out of harm's way. The remaining fat is pushed down under the lower pole of the kidney which is then replaced; the upper pole first.

The ends of the sutures are carried through all the structures of the back at the upper angle of the lumbar incision from within outward and brought out upon the surface where they are tied over a fold of gauze placed lengthwise of the incision[5].

This is readily accomplished with a long curved perineal needle

5. For a detailed description of this technique see article by the author in *American Medicine,* December 28, 1901.

with the eye near its point. When these sutures are tied the kidney is drawn up into its normal position with its upper pole under the ribs. The sutures are tied over the fold of gauze to obviate cutting into the skin and consequent loosening of the suture loop which would permit the kidney to sag away from its attachment.

In the majority of my cases I have employed a folded strip of gauze packed around and under the lower pole of the kidney, bringing the end out at the lower angle of the wound for drainage. Where there is much of the fatty capsule remaining this gauze packing keeps it from crowding between the kidney and muscle and preventing or limiting the area of adhesion, and it also serves to crowd the colon out of the way and furnishes additional support to the kidney, thus lessening the strain on the sustaining sutures. This is by no means essential, however, and when there is not much redundancy of the fatty capsule I do not use it. But I am careful to remove all blood from the wound and when the oozing from the kidney is considerable to use hot salt solution to control it.

The wound is closed by a continued suture of plain catgut, which approximates the upper layer of muscle and the aponeurosis and a subcuticular suture for approximating the skin. The usual dressing of absorbent gauze is applied. When drainage is employed it will be necessary to apply an abundant quantity of gauze to absorb the oozing and this should be renewed after 24 hours. The gauze drain when used is removed at the expiration of 48 hours.

The sustaining sutures are removed on the 20th day and the patient is permitted to get up on the following day. During the period of confinement to bed (three weeks) the patient must be kept strictly on her back if both kidneys have been operated on, being turned only partially on the right side to renew the dressings. If the right kidney only has been fixed the patient may turn on the right side from time to time to rest the back, but never on the opposite side as this would put undue strain on the freshly attached kidney and tend to loosen it.

Some care is necessary after the patient gets about to avoid unusual strains upon the kidney, and it is best that she wear an abdominal belt for several months, as a precautionary measure, and not resume corsets until the belt can be dispensed with.

Up to the present time, I have done 166 consecutive nephropexies on 130 patients, in 36 fixing both kidneys at the same time without a death and without a failure, so far as I have been able to discover. Hence, there is no mortality attending the operation, for what I have done any other careful, painstaking surgeon can do also.

2030 BROADWAY.

## Figures and Tables

**Fig. I. f1:**
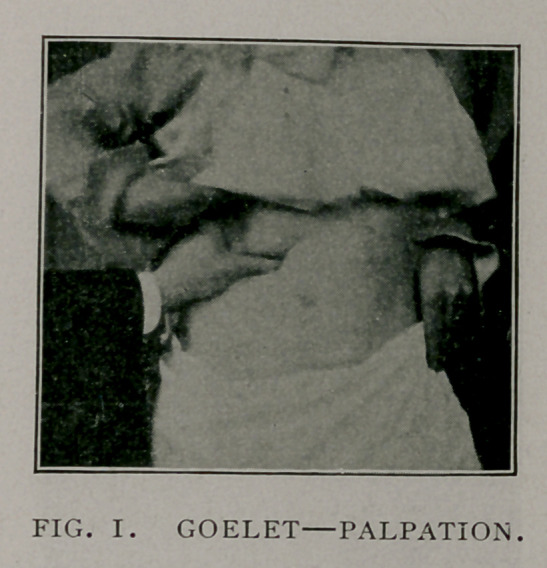


**Fig. 2. f2:**
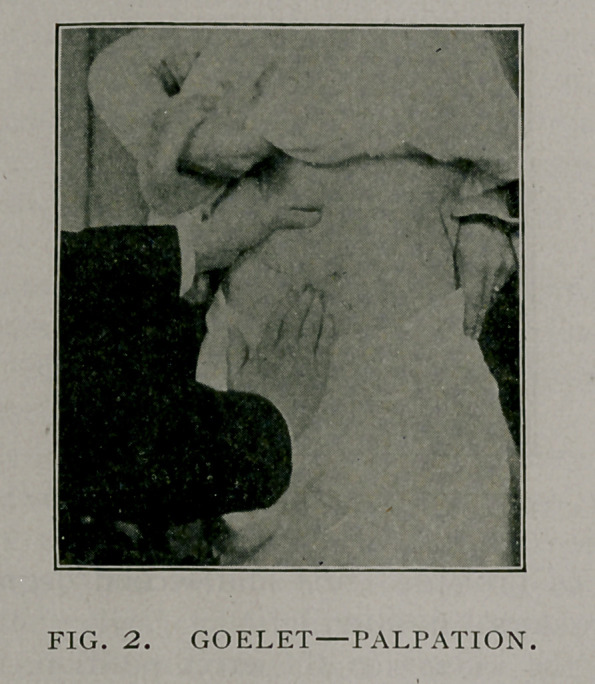


**Fig. 3. f3:**
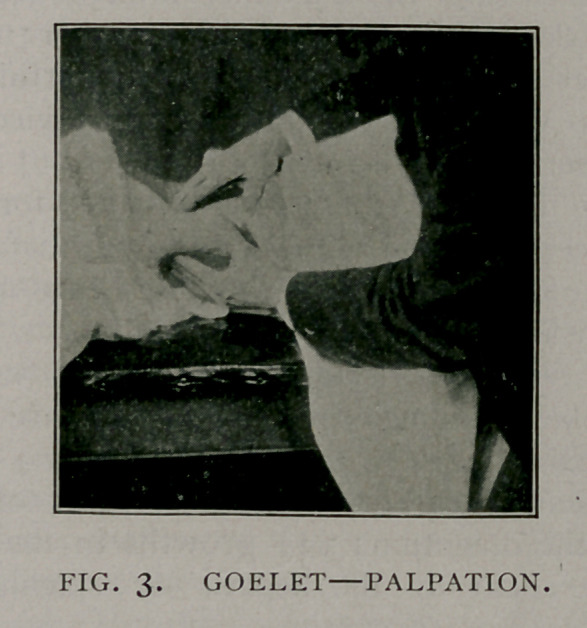


**Fig. 4. f4:**